# Physicochemical Characterization and Thermodynamic Analysis of Avocado Oil Enhanced with *Haematococcus pluvialis* Extract

**DOI:** 10.3390/foods13193184

**Published:** 2024-10-07

**Authors:** Juan G. Báez-González, Melissa M. Gallegos-Garza, Claudia T. Gallardo-Rivera, Mayra Z. Treviño-Garza, Carlos A. Amaya-Guerra, José Rodríguez-Rodríguez, Efraín Obregón-Solís, Eristeo García-Márquez

**Affiliations:** 1 Departamento de Alimentos, Facultad de Ciencias Biológicas, Universidad Autónoma de Nuevo León, Avenida Universidad, Ciudad Universitaria, San Nicolás de los Garza 66455, Nuevo León, Mexico; juan.baezgn@uanl.edu.mx (J.G.B.-G.); melissa.gallegosgrz@uanl.edu.mx (M.M.G.-G.); claudia.gallardorv@uanl.edu.mx (C.T.G.-R.); mayra.trevinogrz@uanl.edu.mx (M.Z.T.-G.); carlos.amayagr@uanl.edu.mx (C.A.A.-G.); 2 Escuela de Ingeniería y Ciencias, Tecnológico de Monterrey, Ave. Eugenio Garza Sada 2501, Monterrey 64849, Nuevo León, Mexico; jrr@tec.mx; 3 Centro de Investigación y Asistencia en Tecnología y Diseño del Estado de Jalisco, Autopista Mty-Aeropuerto Km 10 Parque PIIT, Vía de Innovación 404, Apodaca 66629, Nuevo León, Mexico; esolis@ciatej.mx

**Keywords:** oxidation, avocado oil, *Haematococcus pluvialis*, astaxanthin, sonication, peroxide, activation energy

## Abstract

The consumption of fatty acids offers significant health benefits; however, they are prone to degradation by environmental factors. One method to preserve these fatty acids is the addition of synthetic antioxidants. This study focuses on the determination of peroxide and MDA formation rates at temperatures of 25 °C, 45 °C, and 65 °C. The oxidative stability of cold-pressed avocado oil was evaluated using pure astaxanthin, TBHQ, and *H. pluvialis* extract at concentrations of 100, 500, and 1000 ppm. Kinetic models and thermodynamic analysis were applied to determine the oxidation rate and compare the antioxidant effects of *H. pluvialis* extract with astaxanthin and TBHQ. The Arrhenius model was used to estimate activation energy (Ea), enthalpy, entropy, and free energy. Avocado oil with 500 ppm of *H. pluvialis* extract showed antioxidant effects comparable to TBHQ and pure astaxanthin. The activation energy of plain avocado oil was 40.47 kJ mol^−1^, while with *H. pluvialis* extract, it was 54.35 kJ mol^−1^. These findings suggest that *H. pluvialis* extract offers effective antioxidant properties and could serve as a natural alternative to synthetic antioxidants in food applications, despite the limitations of unprotected astaxanthin.

## 1. Introduction

A correlation has been observed between a diet containing unsaturated fatty acids and its beneficial effects on cardiovascular health, as well as its anti-inflammatory properties [1]. Avocado oil contains approximately 86% unsaturated fatty acids such as oleic, palmitoleic, and linoleic acid (Omega 6) [2]. However, the reactivity of unsaturated fatty acids toward oxygen and small concentrations of water, enhanced by light and temperature, increases their deterioration and formation of peroxides, affecting sensory and nutritional properties, and the shelf life of the final product [2,3]. The routine use of synthetic antioxidants such as BHT, BHA, and TBHQ in foods has prevented the rancidity of oils and fats, although it has been reported that there may be potential risks to human health [4]. The excessive addition or incorrect use of synthetic phenolic antioxidants can result in toxicity and the induction of oxidative stress and endocrine-disrupting effects, which is why more research and stricter regulations have been observed [4,5]. The precise incorporation of synthetic phenolic antioxidants in foods could prevent the underestimation of acceptable intake levels and potential health risks [6]. The findings regarding the potential effects of BHT and BTA [7] in in vivo studies are not recent.

The addition of natural antioxidants such as carotenoids does not require strict regulation, and there are no known harmful effects from excessive consumption of carotenoids. Carotenoids offer beneficial effects: β-carotene has a pro-vitamin A function, while lutein and zeaxanthin constitute macular pigment in the eye and contribute to improvements in cognitive function and cardiovascular health and are strong antioxidants [8,9]. The inclusion of natural carotenoids neutralizes free radicals and slows oxidation reactions in oils. The use of *H. pluvialis* alga extract in the form of free and esterified astaxanthin, with the composition being mainly 5% in free form and 95% esterified, has been examined [10,11]. Astaxanthin in *H. pluvialis* has been found to be a more effective reducing agent compared to vitamin C, vitamin E, and β-carotene, among others [12], in reducing and inhibiting the oxidation rate and the formation of hydrogen peroxides in sunflower oil [13].

Astaxanthin exhibits antioxidant activity that is 10 times more than β-carotene and 100 times more than vitamin E [14]. Its structural stability allows it to neutralize free radicals (hydroxyl, superoxide, and peroxide from the decomposition of hydroperoxides [15]) more effectively. Various studies suggest that the monoesters and diesters of astaxanthin are more stable due to their lipophilic nature compared to the free form [13,14,16]. These esters, abundant in *H. pluvialis*, easily mix with lipophilic food matrices. Vitamin E works by interrupting the lipid oxidation chain but is quickly inactivated when ROS concentrations are high. β-carotene is an effective lipophilic antioxidant, but at high levels, it can become a pro-oxidant, an effect not observed with astaxanthin. Compared to β-carotene and vitamin E, astaxanthin is more stable and more effective as an antioxidant [17], and it is suggested as a potential food supplement [14]. It has been suggested that astaxanthin exhibits greater thermal stability compared to polyunsaturated fatty acids in avocado oil [3,18]. Avocado oil is an ideal vehicle to assess the antioxidant effects of astaxanthin, including its monoesters and diesters at various concentrations. Polyunsaturated fatty acids in avocado oil are prone to oxidation, while astaxanthin not only remains stable but also acts as an antioxidant, protecting against lipid oxidation.

The oxidation rate, Gibbs free energy (${∆G}^{‡}$), enthalpy (${∆H}^{‡}$), entropy **(**${∆S}^{‡}$), temperature effects on the reaction, and the equilibrium constant (k) [19] are essential thermodynamic quantities for predicting reaction behavior, particularly in the formation of peroxides and MDA. Understanding both products is necessary to assess the degradation process of avocado oil and the effects of the antioxidants tested, as well as how temperature influences reaction spontaneity [20,21]. Further studies are needed on the extraction process of astaxanthin and the testing of its extract in various types of edible oils to recommend naturally occurring antioxidants as replacements for synthetic ones. This study, therefore, focuses on the oxidation rate, the effect of temperature on avocado oil, and its thermodynamic parameters using the carotenoids present in *H. pluvialis*.

## 2. Materials and Methods

### 2.1. Materials

The Hass avocados used in this study were purchased from a supermarket in Monterrey, Nuevo León (Mexico). The avocados were processed into avocado oil at the pilot plant of the Noreste unit of CIATEJ (Parque PIIT, Apodaca, Nuevo León, Mexico). The lyophilized powder of *Haematococcus pluvialis* (containing Astaxanthin 3.5%, production date 25 December 2021) was obtained from Nutriherb Biotech Co. (Pharmaceutical Valley, Nanjing, China). Medium-chain triglycerides from coconut oil (MCT oil) were sourced from Bio-nutrients Global, Monterrey, Nuevo León, Mexico. ABTS (2,2′-azinobis (3-etilbenzotiazolín)-6-sulfónico, purity ≥ 98%), DPPH (2,2-diphenyl-1-picrylhydrazyl, purity 98%), potassium persulfate (purity 99.99%), 6-hydroxy-2,5,7,8-tetramethylchroman-2-carboxylic acid (Trolox, 98.5%), tert-butylhydroquinone (TBHQ, purity 97%), 1,1,3,3-tetraethoxypropane (purity ≥ 96%), astaxanthin from Blakeslea trispora HPLC (purity ≥ 97%), and tridecanoic acid (purity ≥ 98%) were purchased from Sigma-Aldrich (Toluca de Lerdo, Mexico). Acetone, ethanol absolute, hexane, soluble starch, and potassium iodine were purchased from CTR Scientific (Monterrey, Nuevo León, Mexico). Additionally, 37% of hydrochloric acid was obtained from DEQ, located in Garcia, Nuevo León, Mexico. Ethyl acetate and 2,2,4-trimethylpentane (isooctane) were obtained from Fermont (Monterrey, Nuevo León, Mexico). Acetic acid and sulfuric acid were purchased from Jalmek (San Nicolas, Nuevo León, Mexico). Sodium thiosulfate was sourced from JT Baker, Fullerton, CA, USA. Thiobarbituric acid was obtained from MP Bio, Santa Ana, CA, USA. All reagents used were of analytical grade.

### 2.2. Oil Extraction and Physicochemical Characterization

Approximately 4.1 kg of avocado pulp was obtained from 6 kg of Hass avocado. The ripeness of the Hass avocado was controlled using the apparent total solid content, and it was 2.25 ± 0.060 °Brix (Anton Paar, Abbemat 300 model, Graz, Austria). The pulp was then dried at 60 °C for 10 h in a dehydrator (Hamilton Beach, Glen Allen, VA, USA). Then, the oil was obtained from the dehydrated avocado pulp and was fed to the cold pressing machine (K39, oil press machine, 950 W, 6–9 kg h^−1^, Guangdong, China). Finally, the oil obtained was subsequently centrifuged at 10,000 rpm for 15 min. Approximately 720 g of cold-pressed fresh avocado oil was obtained (yield of 17.6% *w*/*w*), stored in an amber bottle, and kept at −20 °C until use.

The chemical characteristics evaluated were peroxide value (PV), measured according to the method proposed by Crowe and White [22], with slight modifications, and the acidity index (AI), determined according to the ISO 660:2009 method [23]. The physical characteristics evaluated were relative density (RD) using the pycnometer method according to CODEX and refractive index (RI), determined following the AOCS-CC 7-25 method [24]. A digital refractometer from Anton Paar (Abbemat 300 model, Graz, Austria) was used. All experiments were conducted in triplicate.

### 2.3. Fatty acid Composition

Avocado oil was analyzed using gas chromatography (GC). Briefly, 30 mg of the cold-pressed avocado oil was placed in a 12 mL glass vial, and afterward, the following materials were added: 1 mL of internal standard triundecanoin (4000 ppm) (Saint Louis, MO, USA), 1 mL of hexane, and 2 mL of 10% sulfuric acid. The sample was sealed and heated at 80 °C for 1 h (using an Accublock, Labnet, Edison, NJ, USA). After the reaction, the sample was cooled, and 4 mL of hexane was added and mixed for 1 min. The mixture was left to settle for 10 min, and the organic phase was separated. This process was repeated twice, and organic extracts were combined in a volumetric flask and adjusted to 10 mL. Approximately 2 µL aliquots from each individual sample were injected into a GC equipped with a flame ionization detector (Agilent Technologies, 7890A (GC)/7693(ALS), Santa Clara, CA, USA). The analysis was conducted using the methodology of Forero-Doria et al. [2] and Rozema et al. [25]. The separation was performed using a fused silica capillary column (60 m × 0.25 mm id, 0.20 µm film thickness, Agilent GC, Santa Clara, CA, USA). The initial temperature was set at 100 °C (held for 4 min), which was ramped at 3 °C min^−1^ to a final temperature of 240 °C and held for 15 min. The injector and detector temperatures were set at 225 °C and 285 °C, respectively. Helium was used as the carrier gas at a flow rate of 0.75 mL min^−1^, with a split ratio of 200:1. Peaks were identified using external standards. The components of cold-pressed avocado oil were analyzed as relative percentages with the total area under the peaks representing the total elution.

### 2.4. Extract of H. pluvialis

The organic extracts from *H. pluvialis* were obtained using the methodology proposed by Ruen-ngam et al. [26]. The extraction was conducted using a 1:100 algae powder/acetone ratio in a 250 mL beaker, shielded from light. It was placed on a stirring plate at 400 rpm, with the ultrasonic probe submerged 1 cm deep into the mix. The extraction was carried out at 45 °C, with the ultrasonic energy varying at 40% (2.8 W), 60% (15.3 W), and 80% (20.3 W) for 60 min in an ultrasonic bath (Sonic Ruptor 250, Omni, Kennesaw, Georgia, USA). Subsequently, the solution was filtered, and the sample was concentrated using a rotary evaporator (RV10 digital, IKA, New Hampshire, USA) coupled with a cooling thermostat (Lauda alpha RA8, Marlton, NJ, USA) at 35 °C, 50 rpm, and 500 mbar pressure, as established by Zhao et al. [27]. The following formula was used for calculation:

$$W=\frac{m*C_{p}*(T_{f}-T_{i})}{t}$$

where power is W, m represents g of water, C_p_ is the specific heat capacity of water (4.182 kJ kg^−1^ °C^−1^), T_f_ is the final temperature (°C), T_i_ is the initial temperature (°C), and t represents time (s).

### 2.5. Free Astaxanthin of H. pluvialis Extract by HPLC

The concentration of free astaxanthin was analyzed by HPLC after diluting the dry alga extract in acetone (19 mg mL^−1^) and filtering through a 0.45 μm PTFE filter (Millex-FH, Darmstadt, Germany). The organic solution (*H. pluvialis*) was analyzed using a Hewlett Packard HPLC system with a photodiode array detector. Quantification was performed on a Hypersil ODS C_18_ reversed-phase HPLC column (5 µm particle size, 4 mm × 250 mm, Hewlett Packard, Palo Alto, CA, USA). The gradient elution using A (water), B (methanol), and C (acetone) was set at 0 min 9% A, 76% B, and 15% C; 9 min 5% A, 45% B, and 50% C; 15 min 4% A, 38% B, and 58% C; 17 min 3% A, 27% B, and 70% C; 22 min 3% A, 27% B, and 70% C; 25 min 100% C; 26 min 100% C as described in Orosa et al. [28]. The flow rate was maintained at 1 mL min^−1^, and identification was performed at a wavelength of 480 nm. Data acquisition was subsequently performed using the SCAN method with a mass range (*m*/*z*) of 20 to 550. The identification of components was achieved by comparing the mass spectrum of each compound with the NIST 2008 library, and the proportion of each methyl ester was determined based on the area percentages of each peak as a confirmation of the respective molecules. Astaxanthin (Merk, St. Louis, MO, USA) was utilized as the standard.

### 2.6. Antiradical Activity

The antioxidant activity was determined in a solution of 19 mg of dried extract by mL of ethyl acetate (A_19_). The mixture was vortexed for 10 min, followed by centrifugation at 6500 rpm for 15 min to separate insoluble solids. The sample was filtered through a 0.45 μm PTFE filter (Millex-FH). An aliquot of 300 µL of A_19_ was diluted with 2.7 mL of ethyl acetate, and antiradical activity was measured [29]. The ABTS radical was prepared by mixing potassium persulfate (2.45 mM) and ABTS (7 mM) solutions (1:1, *v*/*v*) and storing the mixture in an amber flask for at least 16 h protected from light. The radical solution was then diluted (2.5 mL in 100 mL of ethanol) [30]. The working solution had an absorbance of 0.700 ± 0.05. From the diluted *H. pluvialis* extract, 300 μL was mixed with 2.7 mL of ABTS• + solution. The mixture was thoroughly mixed, and absorbance was measured 7 min later at 734 nm using a Spectronic UV–visible spectrophotometer (Genesys 5, Thermo Fisher Scientific, Waltham, MA, USA).

The antiradical activity was also measured using the DPPH (2,2-diphenyl-1-picrylhydrazyl) discoloration test. A 0.1 mM DPPH stock solution was prepared and adjusted to an absorbance of 1 with ethanol [30,31]. Then, 750 µL of the working solution was mixed with 2.25 mL of DPPH, and after 90 min, the absorbance was measured at 517 nm [31]. Trolox solution was used to generate a standard curve for both methods, and the results are expressed as µM Trolox equivalents per gram of oil. The analysis of ABTS and DPPH was performed in triplicate.

### 2.7. Stability Cold-Pressed Avocado Oil with H. pluvialis Extract

Oil samples with extract and control samples were stored for 36 days at 25 °C, 45 °C, and 65 °C, protected from light. During storage, peroxide levels (meq O_2_ kg^−1^) and secondary oxidation products (TBARSs) were measured. The dry *H. pluvialis* extract was added to avocado oil (A) at concentrations of 100 ppm (A_100_), 500 ppm (A_500_), and 1000 ppm (A_1000_). Additionally, three control samples were prepared: one with 400 ppm astaxanthin (A_AST_), one with 80 ppm TBHQ (A_TBHQ_), and one without any antioxidant (A_WA_). All samples were gently mixed until fully dissolved.

### 2.8. Peroxide Value

The PV was evaluated and expressed as milliequivalents of active oxygen peroxide per kilogram of oil (meq O_2_ kg^−1^), as proposed by Crowe and White [22]. In short, 0.5 g of oil was placed in an Erlenmeyer flask, to which 3 mL of acetic acid–isooctane (3:2) was added. The mixture was stirred, and then 50 μL of the saturated potassium iodide solution was introduced. After stirring, the solution could stand. Subsequently, 3 mL of oxygen-free distilled water (freshly boiled) was added, followed by the addition of 200 μL of a 1% starch solution.

Finally, the peroxide value (PV) was quantified using a sodium thiosulfate solution. The peroxide value was calculated using the following equation:

$$PV=\frac{N*V*1000}{g\;sample}$$

where N is 0.01 N sodium thiosulfate, and V is the volume of sodium thiosulfate. The analysis was conducted in triplicate.

### 2.9. Thiobarbituric Acid Value (TBARS Method)

Malondialdehyde (MDA) analysis in oil samples was conducted following the methodology proposed by Poyato et al. [32]. In summary, 2.5 mL of distilled water was added to 910 µL of oil, and the sample was vigorously mixed for 2 min using a vortex. The sample was centrifuged at 5000 rpm for 5 min to separate the phases. Next, 2 mL of the supernatant was transferred to a test tube, and 2 mL of TBA reagent (46 mM) was added. The mixture was heated in a water bath at 100 °C for 35 min and then cooled in an ice bath for 10 min. After centrifugation at 6000 rpm for 20 min, the absorbance of the supernatant was measured at 532 nm using a spectrophotometer (Thermo Scientific Genesys 10S UV-Vis). MDA concentrations were calculated using a calibration curve prepared with 1,1,3,3-tetraethoxypropane (TEP) ranging from 0.1 to 2.4 mg kg^−1^. The results are expressed as mg MDA per kilogram of oil (mg kg^−1^), and the final value represents the average of three independent analyses.

### 2.10. Rate Peroxide and Malondialdehyde

The increase in peroxide was calculated as the average of the specific repetitions at each temperature evaluated over the 36-day storage period. The Arrhenius equation was used to calculate the activation energy, which is the energy barrier that must be overcome for fatty acids to transform into peroxides, the molecules monitored during the trial [19,33].

$$k=A_{b}exp\frac{\left(-E_{a}\right)}{RT}$$

The linear form of the equation is as follows:

$$ln\;k=-\left(\frac{Ea}{R}\right)\frac{1}{T}+InA_{b}$$

where *k* is the rate constant (meq O_2_ kg^-1^ oil h^-1^), A_b_ is the pre-exponential factor, *Ea* represents the activation energy (kJ mol^−1^), R is the universal gas constant 8.3145 × 10^−3^ (kJ mol^−1^ K^−1^), and T is the absolute temperature (K).

### 2.11. Thermodynamic Study

The formation of peroxides depends on the free energy absorbed in the system, occurring during the reaction shift between unsaturated fatty acids (reactants) and peroxide formation. Calculating thermodynamic parameters is crucial for estimating the amount of energy required for the chemical process to occur.

Enthalpy (${∆H}^{‡}$) and entropy (${∆S}^{‡}$) were derived from the Eyring equation as follows:

$$ln\frac{k}{T}=-\frac{{∆H}^{‡}}{R}*\frac{1}{T}+ln\frac{k_{B}}{h}+\frac{{∆S}^{‡}}{R}$$

where *k_B_* is the Boltzmann constant (1.38065 × 10^−23^ J K^−1^), and h is the Planck constant (6.626070 × 10^−34^ J s).

Using the fundamental equation of thermodynamics, the Gibbs free energy (${∆G}^{‡}$, kJ mol^−1^) was calculated utilizing the enthalpy change, the universal gas constant, and the absolute temperature [34] as follows:

$${∆G}^{‡}={∆H}^{‡}-{∆S}^{‡}$$

The data were calculated considering the average of three replicates.

### 2.12. Statistical Analysis

Analysis was conducted using SPSS 23.0 (SPSS Inc, Chicago, IL, USA). All experiments were performed in triplicate, and the results are reported as mean ± standard deviation (SD). Differences between samples were determined using one-way and two-way ANOVA at the 5% level of significance (*p* < 0.05).

## 3. Results and Discussion

### 3.1. Avocado Oil

#### 3.1.1. Physicochemical Characterization

Avocado oil mainly contains fatty acids like oleic acid, linoleic acid, and palmitic acid. The oil obtained through cold pressing was characterized by quantifying hydroperoxides, acidity value, and refractive index. These values were then compared with the minimum parameters established for cold-pressed and refined avocado oils intended for human consumption, as set by Mexican regulations [35]. The hydroperoxide value obtained was 9.45 ± 1.011 meq O_2_ kg^−1^ of oil, 2.31 ± 0.10% free fatty acids, a refractive index of 1.46750 ± 0.0005, and a density of 0.910 g mL^−1^. The physicochemical characterization data of the cold-pressed oil generally did not exceed the established limit values of 10.0 meq O_2_ kg^−1^ of oil, 5.0% free fatty acids (as oleic acid), and a density range of 0.91–0.92 g/cm^3^. Thus, the oil was suitable for use in the oxidative stability study. These findings are relevant for ensuring the quality and safety of avocado oil intended for human consumption, as per regulatory standards [36]. Kilic-Buyukkurt et al. [37] and Poyato et al. [32] reported a hydroperoxide value of 10.19 ± 0.84 meq O_2_ kg^−1^ in cold-pressed avocado oil, while Prescha et al. [38] obtained a value of 9.55 meq O_2_ kg^−1^. These findings further confirm that cold pressing is a suitable method for obtaining oils with low levels of peroxides, which is critical for maintaining the oil’s oxidative stability and nutritional value.

#### 3.1.2. Fatty Acid Composition in Avocado Oil

The fatty acid profile of cold-pressed avocado oil, analyzed by gas chromatography, revealed 17.2% saturated fatty acids,71.80% monounsaturated fatty acids, and 11.0% polyunsaturated fatty acids. The ratio of monounsaturated to polyunsaturated fatty acids was 6.5:1, while the ratio of monounsaturated to saturated fatty acids was 4.17:1. Table 1 outlines the relative percentages of the main fatty acids present in avocado oil. Santana et al. [39] reported 22.2% saturated fatty acids, 66.1% monounsaturated fatty acids, and 12.0% polyunsaturated fatty acids in avocado oil. Similarly, Berasategi et al. [40] documented 19.3% saturated fatty acids (palmitic acid: 18.7%), 68.4% monounsaturated fatty acids (palmitoleic acid: 7.9%, oleic acid: 54.4%, and vaccenic acid: 5.9%), and 11.75% polyunsaturated fatty acids (linoleic acid: 10.9%). Wang et al. [41] reported a combined 66.2% of total monounsaturated fatty acids (oleic acid and vaccenic acid). The results of the fatty acid profile obtained by GC that we present coincide with the information previously reported by other authors.

#### 3.1.3. *H. pluvialis* Extract Characterization and HPLC

*Haematococcus pluvialis*, like many other algae, has a cell wall composed of polysaccharides, proteins, and mannan polymer, in addition to astaxanthin. These components provide structural support and protection to the cell. Within the cell, high concentrations of astaxanthin accumulate in lipid microbodies within the cytoplasm [42]. The extent of astaxanthin accumulation directly correlates with the intensity of the cell’s coloration. Astaxanthin has been utilized as a nutritional supplement, demonstrating efficacy in preventing various chronic degenerative diseases [12]. However, due to the composition of its cell wall and the lipid–astaxanthin combination in its core, the leaching of astaxanthin is challenging, requiring a significant amount of energy to extract this antioxidant due to the fact that it is a mixture of different structures of astaxanthin [42,43]. The concentration of free astaxanthin in the extract was directly influenced by the level of sonic energy applied.

With higher energy power, the concentration of free astaxanthin and antioxidant activity, which were quantified through DPPH and ABTS assays, increased. Each data point is included in Table 2. The observed variations in free astaxanthin were significantly influenced by the energy supplied (sonication) during the astaxanthin extraction process.

The quantification of free astaxanthin by HPLC (Table 2) proportionally increased with higher sonic power. A power level of 20.2 W exhibited the highest concentration of astaxanthin, reaching 8.26 mg g^−1^ of the *H. pluvialis* dry extract. This finding is similar to that of Ruiz-Domínguez et al. [44], who reported 6.82 ± 0.19 mg g^−1^ of free astaxanthin when quantifying the carotenoids of *H. pluvialis*. Similarly, when examining the ratio of individual antioxidant activity relative to the amount of astaxanthin leached as a function of the supplied energy power, an average value of 1.34 was observed when the energy rates were 2.8 and 15.3 W. However, the ratio was less than one when the energy was high. This implies that antioxidant activity is directly proportional to the concentration of astaxanthin (μmol eq. Trolox mg^−1^ astaxanthin). It is evident that due to its cell wall complexity, *H. pluvialis* is able to retain astaxanthin when the supplied energy is at 2.8 W. The leaching of astaxanthin increased by approximately 1.34 times compared to when the sonic energy was low, and an increase of antioxidant activity was observed when the energy was greater than 15.3 W.

It has been noted that increasing sonication may be sufficient to break down the strong bonds and bioactive compounds (total phenolics, flavonoids, and tannins), which increases experimental variability [45,46]. This effect was reported by Annegowda et al. [46]. However, in the range of sonic energy applied, the extraction of esterified compounds from *H. pluvialis* cells was enhanced by sonication, and the previously described effect was not observed. Another important consideration is the polarity and saturation of the solvent [47] used in this study. Additionally, it has been reported that there could be interference during the quantification of carotenoids in *H. pluvialis* (400–500 nm) in the presence of the DPPH reagent, whose maximum absorption wavelength is 517 nm [48]. The results obtained are consistent with those of Goiris et al. [49], who determined an antioxidant activity of 5.71 ± 0.12 µmol eq. Trolox g^−1^ of dried algae using the ABTS method in ethanol/water extracts of *H. pluvialis*.

**Figure 1:**
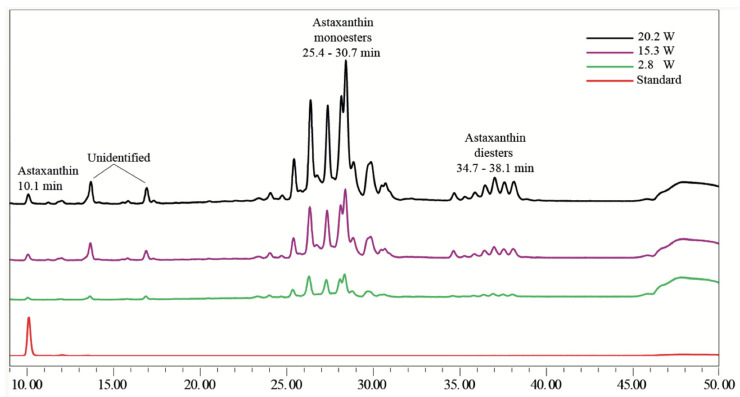
HPLC chromatogram (DAD, 480 nm) of molecules in *H. pluvialis* extracts, including astaxanthin, monoesters, and diesters, obtained using acetone as the solvent under three different sonication levels, along with the astaxanthin standard.

The different molecules in the organic acetone extract of *H. pluvialis*, analyzed by HPLC, are shown in Figure 1. Three regions corresponding to carotenoids (free form, monoesters, and diesters) were identified. Free astaxanthin appeared at a retention time of 10.1 min, constituting approximately 5% of the total relative concentration. Notably, around 70% of the astaxanthin observed in the chromatogram was identified as astaxanthin monoester, eluting between 25 and 30 min, while 25% was astaxanthin diester, which was detected between 35 and 39 min. These findings are consistent with those reported by Todorović et al. [10]. Furthermore, the HPLC analysis revealed that the majority of astaxanthin was present in esterified form, with higher levels observed at 20.2 W.

#### 3.1.4. Avocado Oil with *Haematococcus pluvialis* Extract

Lipid peroxidation has been linked to an increase in diseases such as Alzheimer’s [50]. The stability of fresh oils with a higher proportion of fatty acids was preserved during the study using the extract of *H. pluvialis*. This finding allows us to hypothesize that with the prolonged stability of fatty acids, they can be consumed more safely, potentially leading to better health outcomes [2].

Various studies have reported that freshly extracted natural oils contain antioxidant compounds that help prolong shelf life by providing antioxidant protection [2,3,51]. The structure of astaxanthin allows it to efficiently neutralize free radicals, such as hydroxyl (OH^−^), superoxide anion (O_2_^−^), and hydrogen peroxide (H_2_O_2_), by donating electrons without losing stability. This is due to the presence of conjugated double bonds in its structure [52]. However, the shelf life of products is typically shorter. While synthetic antioxidants have been used to protect oils high in unsaturated fatty acids, their use is regulated [4]. The antioxidant activity of astaxanthin as a natural antioxidant is 10 times more than zeaxanthin, lutein, canthaxanthin, and β-carotene and 100 times better than α-tocopherol, and it can reduce the rate of oxidation [14,43,53]. However, at high concentrations, these molecules increase the oxidant effect in the oil, and temperature is also a limiting factor, making it crucial to determine optimal concentrations and maintain controlled temperature during shelf life.

**Figure 2:**
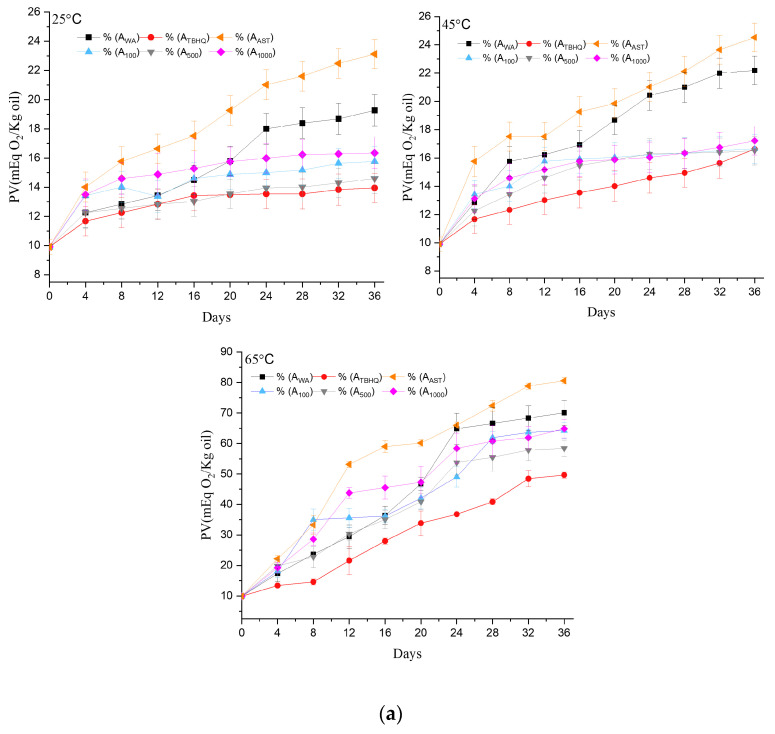

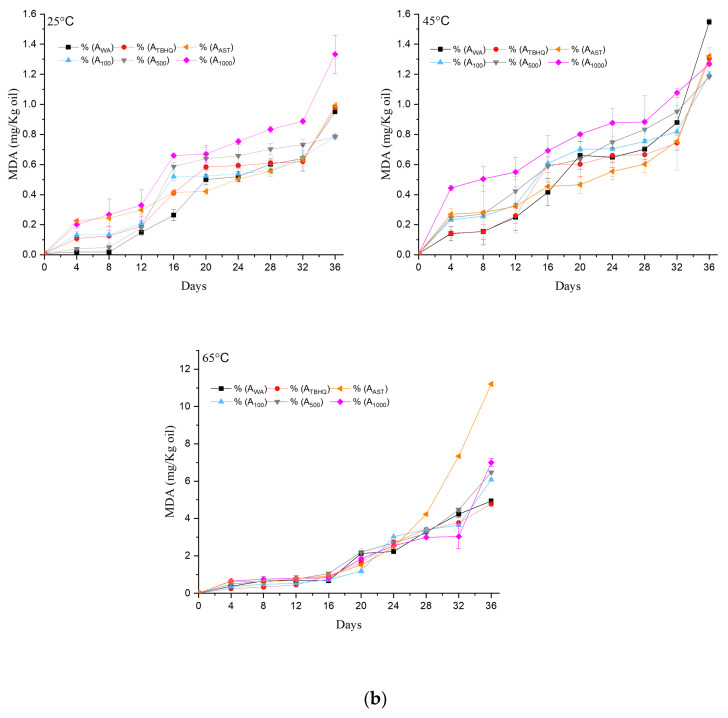
(**a**) The formation of peroxides from unsaturated fatty acids in avocado oil, evaluated during storage at 25 °C, 45 °C, and 65 °C, using different concentrations of astaxanthin extract, pure astaxanthin, and TBHQ as a positive control, along with a sample without any antioxidants. The reported values represent the mean of three independent tests. (**b**) The formation of malondialdehyde from unsaturated fatty acids in avocado oil, evaluated during storage at 25 °C, 45 °C, and 65 °C, using different concentrations of astaxanthin extract, pure astaxanthin, and TBHQ as a positive control, along with a sample without any antioxidants. The reported values represent the mean of three independent tests.

The monitoring of peroxide formation at 25 °C and 45 °C in the untreated avocado oil sample (A_WA_) showed similar levels at the end of the study (19.0 and 22.0 meq O_2_ kg^−1^ oil, respectively), with no significant difference observed. However, at 65 °C, there was a notable increase in peroxide formation to 70.0 meq O_2_ kg^−1^ oil from the initial concentration of 10 meq O_2_ kg^−1^ oil (Figure 2a). In contrast, the positive control (A_TBHQ_) exhibited less peroxide formation compared to the untreated oil. The maximum concentrations of peroxides formed at 35 days were 14.0 (25 °C), 16.5 (45 °C), and 52 (65 °C) meq O_2_ kg^−1^ oil, relative to the initial concentration. On the other hand, the addition of 400 ppm of pure astaxanthin per kg of cold-pressed oil (A_AST_) increased peroxide formation by 21.0% at 25 °C, 11.4% at 45 °C, and 17.1% at 65 °C compared to the sample without antioxidant. This suggests that pure astaxanthin exhibited an oxidizing behavior rather than acting as an antioxidant in avocado oil. Yang et al. [27] reported that free astaxanthin is susceptible to degradation when subjected to storage at temperatures ranging from 25 °C to 50 °C, making it more susceptible to degradation compared to esterified astaxanthins. Moreover, the degradation rate increases with higher storage temperatures [54,55]. Pure carotenoids have the opposite effect compared to astaxanthin monoester mixtures, which reduce the formation of peroxides in avocado oil [56]. However, Espinaco et al. [57] evaluated the oxidation rate of chia oil stabilized with 100 ppm of astaxanthin and observed that the control chia oil produced twice as many peroxides compared to the astaxanthin-stabilized sample when stored at 25 °C for 35 days.

The formation of peroxides in avocado oil stored at 25 °C and 45 °C, with the addition of A_100_, A_500_, and A_1000_ ppm of the organic astaxanthin extract, was less than the control sample (A_WA_). In general, the addition of 100 and 1000 ppm of *H. pluvialis* extract during storage at 25 °C for 34 days resulted in a 19% decrease in the oxidation rate compared to the A_WA_ sample. Similarly, at 45 °C, the oxidation rate was reduced by 29.5% (Figure 2a). The average concentration of peroxides in the samples with astaxanthin was 6 meq O_2_ kg^−1^ of oil higher than that in the control (A_WS_) sample, as the presence of astaxanthin in ester and diester forms contributed to mitigating peroxide formation. This suggests that at temperatures equal to or below 45 °C, the organic astaxanthin extract effectively protects natural avocado oil, even though it contains a high concentration of unsaturated fatty acids. No significant difference in peroxide formation was observed with the addition of 100, 500, and 1000 ppm of *H. pluvialis* under storage conditions of 25 °C and 45 °C, but for the sample stored at 65 °C, peroxide formation increased to 72 meq O_2_ kg^−1^ compared to 69 meq O_2_ kg^−1^ in the A_WA_ sample.

The findings presented here are similar to those reported by Wang et al. [41], who investigated the stability of sunflower oil by adding 200, 600, and 1000 ppm of *H. pluvialis* extract and observed no significant difference in peroxide formation. The addition of *H. pluvialis* extract at concentrations ranging from 100 to 1000 ppm showed no significant difference in peroxide formation, which is attributed to the stability of esterified astaxanthin, as reported by Rao et al. [58]. Carotenoids protect against and reduce oxidation rates. Crecedo-Cruz et al. [13] used chipotle chili oleoresin in avocado oil at a ratio of 1:3 (*w/v*) and observed that peroxide formation during 28 days of storage was less than the sample without carotenoids.

The peroxide formation rates using *H. pluvialis* (at concentrations A_100_, A_500_, and A_1000_) were comparable to those using TBHQ during storage below 45 °C; however, during storage at 65 °C, the samples stabilized with *H. pluvialis* showed a 38% increase in peroxide formation compared to the sample stabilized with 80 ppm TBHQ (A_TBHQ_). The increase in temperature substantially enhanced peroxide formation, despite the addition of TBHQ, which resulted in 50 meq O_2_ kg^−1^ oil.

The oxidation of unsaturated oils can lead to lipid deterioration during processing or storage. Malondialdehyde (MDA) is a byproduct used to assess the deterioration of unsaturated fatty acids [32]. The quantification of MDA through the TBARS (thiobarbituric acid reactive substance) colorimetric reaction revealed that, at 25 °C, the avocado oil sample supplemented with 1000 ppm of the *H. pluvialis* extract exhibited a higher concentration of MDA, indicating a pro-oxidant effect. As shown in Figure 2b, the specific temperatures of 25 °C, 45 °C, and 65 °C individually increased the concentrations of peroxides and malondialdehyde, characteristic products of oil oxidation. Vandemoortele [59] identified that malondialdehyde is primarily formed in oils through an irreversible aldol self-condensation reaction, suggesting its role in advanced oxidation. Frankel [60] identified peroxides as primary oxidation products, serving as precursors to malondialdehyde, a key component in advanced oxidation. Guillén and Ruiz [61] revealed that peroxides are reactive in the formation of malondialdehyde, highlighting a connection between primary and advanced oxidation. Similarly, oils without antioxidants (A_WA_) at 45 °C and avocado oil with astaxanthin pure at 65 °C showed increased MDA concentrations, indicating lipid deterioration—a key index of oxidative rancidity in oils rich in polyunsaturated fatty acids [62] as a fish oil. In line with various studies suggesting that peroxide formation is the primary product of unsaturated fatty acid degradation and that the advanced reaction process involves the formation of MDA, we found that the critical point to address is the reduction in the initial peroxide formation. Based on our findings, the advanced reaction—MDA formation—is crucial within the first eight days of storage to prevent rancidity in the oils intended for food consumption.

#### 3.1.5. Kinetics and Thermodynamics

The formation of peroxide in avocado oil stored at temperatures of 25 °C, 45 °C, and 65 °C was assessed to estimate the reaction order. The formation rate constant (*k*) of peroxides was obtained using the zero-order kinetic model [3], and this information was used to calculate the activation energy (*Ea*) using the Arrhenius equation [19]. Finally, the formation rate was used to obtain thermodynamic parameters, ΔH^‡^ and ΔS^‡^, using the Eyring equation [20]. The activation energy data, obtained from the Arrhenius equation, were used to verify the stability of the oil by comparing it with pure astaxanthin, astaxanthin extract, and TBHQ as a synthetic phenolic antioxidant.

The oxidation rate of oil, represented by the constant *k* (Table 3), increases in the absence of antioxidants (A_WA_), as previously described by Zhan et al. [20]. However, the addition of free astaxanthin or *H. pluvialis* extract in avocado oil significantly reduced the oxidation rate. Similar results have been reported by Cheung et al. [21] and Ahn et al. [63]. Different investigations have reported zero-order kinetic production or degradation. Zhang et al. [64] described that the linoleic acid content in hybridized hazelnut oil fits a zero-order kinetic oxidation model. Similarly, Conte et al. [65] monitored conjugated trienes, hexanal, % pyropheophytin a, pheophytin, and UV absorption at 270 nm as indicators to track the oxidation rate in extra virgin olive oil stored in the dark at 25 °C, 40 °C, 50 °C, and 60 °C, and they observed that the increase in these indicators follows a zero-order kinetic model. Finally, Gómez-Alonso et al. [66] studied the autoxidation of olive oil triacylglycerols protected from light between 25 °C and 75 °C, in the absence of pro-oxidants and antioxidants, and observed that the peroxide value and UV light absorption at 270 nm correlated with a zero-order kinetic model.

The higher the activation energy (*Ea*), the more energy the molecules need to initiate the oxidation reaction, resulting in greater stability of the oil against oxidation, especially at lower storage temperatures [33], and when adding antioxidants to edible oils, *Ea* increases compared to the control [67]. The addition of astaxanthin (A_500_) to avocado oil resulted in an activation energy value of 54.35 kJ mol^−1^, which is similar to that of TBHQ (53.14 kJ mol^−1^). For astaxanthin (A_100_), the value was 51.85 kJ mol^−1^. In contrast, for astaxanthin (A_1000_) and pure astaxanthin (A_AST_), the *Ea* values were 49.53 kJ mol^−1^ and 35.64 kJ mol^−1^, respectively. Ceredero-Cruz et al. [13] added chipotle chili oleoresin to cold-pressed avocado oil, and the activation energy calculated for the formation of peroxides was 34.6 kJ mol^−1^. As shown in Table 3, the Ea calculated for avocado oil was 40.48 kJ mol^−1^, which was slightly different. Piedrahita et al. [33] added rosemary extract to choibá oil and observed that the activation energy increased by around 60% compared to the control oil. Pu and Sathivel [56] determined *Ea* in the control flaxseed oil and samples with astaxanthin and observed changes in *Ea*, with values of 51.07 and 65.84 kJ mol^−1^, respectively. The higher the *Ea* value, the more energy the molecules need to start the oxidation reaction, which results in greater stability to oxidation when the storage temperature is lower.

The Gibbs energy change was positive, with values ranging from 84.27 to 87.22 KJ mol^−1^ at 25 °C, 87.72 to 89.90 at 45 °C, and finally, 91.16 to 92.36 KJ mol^−1^ at 65 °C, indicating that the reaction is non-spontaneous at these temperatures [34]. The degradation process of unsaturated fatty acids into peroxides requires energy in the system under constant pressure and temperature conditions. The enthalpy change was also positive, suggesting that the reaction is endothermic. Additionally, the entropy change of the system was negative, implying a decrease in disorder. Therefore, the transformation of unsaturated fatty acids into peroxides requires an input of energy, contributing to the deterioration process of avocado oil. The determined thermodynamic parameters significantly impacted the stability of unsaturated fatty acids in avocado oil. Low enthalpy and ${\Delta G}^{‡}$ values indicate that the oxidation reaction is thermodynamically favorable, leading to higher susceptibility to oxidation (Table 3). An energy barrier of ${\Delta G}^{‡}$ was observed when the astaxanthin concentration was A_500_ within the temperature range studied. At higher and lower A_500_ values, the energy barrier was reduced, allowing oxidation to occur more easily. This resulted in greater thermal instability, particularly at elevated temperatures, potentially leading to oil degradation through the formation of peroxides and other oxidation products.

## 4. Conclusions

The addition of *Haematococcus pluvialis* extract in cold-pressed avocado oil (a rich source of unsaturated fatty acids) effectively controls peroxide formation when stored at or below 45 °C, adhering to established physicochemical standards. This makes it a promising alternative to synthetic antioxidants, as it demonstrates antioxidant properties. However, at 65 °C, its efficacy declined, highlighting the importance of optimal temperature control and concentration to prevent oxidation in oils rich in unsaturated fatty acids. The fatty acid profile of cold-pressed avocado oil, analyzed by gas chromatography, is consistent with previous studies, confirming its compositional stability. Our findings suggest that the *H. pluvialis*-derived astaxanthin extract has similar antioxidant effects to pure astaxanthin, indicating that sonication can be offset by subsequent purification processes. Additionally, the *H. pluvialis* extract significantly increases the oxidative stability of avocado oil by raising activation energy values, comparable to TBHQ. Thermodynamic parameters, such as positive Gibbs energy and enthalpy, indicate that the oxidation of unsaturated fatty acids is a non-spontaneous, endothermic process requiring energy input. The negative entropy suggests a decrease in disorder during peroxide formation. Overall, the extract stabilized avocado oil effectively at temperatures up to 45 °C, though increased peroxide formation occurred at higher temperatures. This emphasizes the importance of controlled storage conditions to optimize antioxidant efficacy. Environmental factors are crucial in hydrogen peroxide formation, and this study serves as a guide for food technologists to determine optimal natural antioxidant concentrations for reducing rancidity in edible oils and improving human health.

## Figures and Tables

**Table 1:** Relative concentrations of the main fatty acids in avocado oil determined by gas chromatography.

Total Fatty Acids	Fatty Acid	Relative Percentage
Polyunsaturated fatty acids		
	Linoleic acid	11.00 ± 0.35
Monounsaturated fatty acids		71.80
	Palmitoleic acid	14.25 ± 0.17
	Oleic acid	48.10 ± 0.11
	Vaccenic acid	9.54 ± 0.21
Saturated fatty acid		
	Palmitic acid	17.20 ± 0.34

**Table 2:** Astaxanthin concentration (HPLC) and antioxidant activity of *H. pluvialis* (dry weight; DW) as a function of levels of supplied energy.

Power (W)	Free Astaxanthin (mg g^−1^)	DPPH (μmol eq. Trolox g^−1^ DW)	ABTS (μmol eq. Trolox g^−1^ DW)
2.80	3.53 ± 0.07 ^a^	3.50 ± 0.64 ^a^	4.95 ± 0.04 ^a^
15.3	4.71 ± 0.03 ^b^	6.21 ± 0.91 ^b^	6.21 ± 0.59 ^b^
20.2	8.26 ± 2.42 ^c^	6.81 ± 1.45 ^b^	7.30 ± 0.26 ^c^

The values presented are averages of three repetitions with their respective standard deviations. Different superscript letters within columns indicate statistically significant (*p* < 0.05) differences.

**Table 3:** Primary oxidation products: kinetic and thermodynamic parameters of avocado oil with different treatments based on the Arrhenius and Eyring equations.

Kinetics and Thermodynamics	Oil Samples					
	A_WA_	A_TBHQ_	A_100_	A_500_	A_1000_	A_AST_
${\Delta H}^{‡}$ (kJ mol^−1^)	37.84	50.51	49.22	51.72	46.89	33.01
${\Delta S}^{‡}$ (kJ mol^−1^ K^−1^)	−157.68	−123.83	−126.80	−119.13	−133.56	−172.04
${\Delta G}^{‡}\;$25 °C (kJ mol^−1^)	84.83	87.41	87.01	87.22	86.70	84.27
${\Delta G}^{‡}$ 45 °C (kJ mol^−1^)	87.98	89.89	89.54	89.60	89.37	87.72
${\Delta G}^{‡}$65 °C (kJ mol^−1^)	91.13	92.36	92.08	91.99	92.04	91.16
*Ea* (Kj mol^−1^)	40.47	53.14	51.85	54.35	49.53	35.64
*k* (meq O_2_ kg^−1^ oil h^−1^) 25 °C	10.84 × 10^−3^	3.78 × 10^−3^	4.99 × 10^−3^	4.26 × 10^−3^	5.64 × 10^−3^	14.02 × 10^−3^
*k* (meq O_2_ kg^−1^ oil h^−1^) 45 °C	13.57 × 10^−3^	6.72 × 10^−3^	6.01 × 10^−3^	6.84 × 10^−3^	6.46 × 10^−3^	13.93 × 10^−3^
*k* (meq O_2_ kg^−1^ oil h^−1^) 65 °C	77.59 × 10^−3^	49.58 × 10^−3^	62.37 × 10^−3^	59.42 × 10^−3^	63.03 × 10^−3^	80.02 × 10^−3^
R^2^	0.81	0.88	0.78	0.85	0.76	0.71

## Data Availability

The original contributions presented in the study are included in the article, further inquiries can be directed to the corresponding author.
